# Fractional Excretion of Sodium and Urea are Useful Tools in the Evaluation of AKI: PRO

**DOI:** 10.34067/KID.0002492022

**Published:** 2022-09-20

**Authors:** Abdurrahman Hamadah, Kamel Gharaibeh

**Affiliations:** 1 Section of Nephrology, St. Luke's Hospital, Duluth, Minnesota; 2 University of Maryland School of Medicine, Baltimore, Maryland; 3 Faculty of Medicine, Al-Quds University, Jerusalem, Israel

**Keywords:** clinical nephrology, AKI, sodium, urea

The idea of using a spot urinary measure to assess whether AKI was a transient state due to decreased perfusion (prerenal AKI) versus a persistent kidney injury (acute tubular necrosis [ATN] or intrinsic AKI) was popularized in the 1960’s–1970’s. In 1976, in his seminal study published in *JAMA*,^1^ Espinel evaluated the use of fractional excretion of sodium (FENa) in differentiating prerenal AKI versus ATN. FENa represents the ratio of excreted-to-filtered sodium and is a measure of the state of sodium reabsorption by the tubules.

In the original study by Espinel, he studied 17 patients and found that the mean FENa level in prerenal patients was 0.64 (almost all patients were 1% or below) versus 3.77 for the ATN group (almost all patients in this group had values of 3% or above). This study was particularly selective; all 17 patients were oliguric; and patients with urinary obstruction, GN, CKD, and those on diuretics were excluded. Notably in this study, patients were considered prerenal if they responded promptly to intervention, such as patients with hypovolemia responding to intravenous fluids. Patients with heart failure were included in the prerenal category and were described as improved if heart failure improved with diuretics and other measures. Patients were considered to have ATN if oliguria persisted for >1 week despite corrective measures and if confirmed by historiology.

In a subsequent study in 1978 by Miller *et al.*,^2^ a prospective evaluation of 102 patients looked at multiple urinary indices, including FENa, to differentiate the type of AKI. In this study, a FENa of <1% was observed in 27 of 30 (90%) prerenal patients and only in one of 24 (4%) patients with oliguric ATN. A FENa of <1% was also encountered in 10% of patients with nonoliguric ATN. They noted that FENa is a reliable index for the early differentiation of prerenal AKI from ATN and also noted that patients who were nonoliguric frequently had values for diagnostic indices intermediate between those of patients with prerenal AKI and oliguric ATN and concluded that FENa is predominantly of value in oliguric patients. Multiple subsequent studies also showed a good value for FENa in differentiating prerenal AKI from ATN.^3–5^ The abovementioned studies, however, did not include patients with CKD or patients on diuretics. In addition, some reports found that FENa <1% can be seen in states other than prerenal AKI (such as pigment nephropathy) and >1% can be seen in states other than ATN (such as with diuretic use).^6,7^ These situations, however, are relatively uncommon causes of AKI in the hospital because most of them are due to prerenal AKI and ATN.

The fact that FENa was noted to be altered in the presence of a diuretic effect on tubular absorption of sodium, led to the investigation of fractional excretion of urea (FEUrea). This is based on the notion that urea absorption is largely in the proximal tubule and less affected by downstream effects of loop and thiazide diuretics. In the initial study to examine FEUrea, Kaplan and Kohn in 1992^8^ prospectively examined FEUrea in six patients who had received diuretics and showed a FENa of >1%, but a FEUrea of <35%. They then retrospectively analyzed data from 87 urine samples of 40 patients who had both FENa and FEUrea present and noted that in 40 samples where FEUrea was <35% despite a FENa >1%, 39 of 40 patients had received furosemide before obtaining the samples. Ten years later, Carvounis *et al.*^9^ prospectively investigated the ability of FEUrea to differentiate between prerenal and intrinsic AKI in 102 episodes of AKI. They excluded patients with GN, urinary obstruction, and interstitial nephritis. They found that although 92% of patients with prerenal AKI had FENa <1%, only 48% of patients with prerenal AKI who were on diuretics had FENa <1%. In this same group (prerenal AKI on diuretics), 89% patients were found to have FEUrea <35%. They also found that a FEUrea of <35% had a positive predictive value of 98% for diagnosis of prerenal disease. They showed that a low FEUrea of <35% was more sensitive and specific than FENa in differentiating between prerenal and intrinsic AKI, especially in the presence of diuretic therapy. Other subsequent studies have also demonstrated the ability of FEUrea to differentiate between prerenal and intrinsic AKI and in multiple settings.^10,11^

These findings, however, were not seen across all studies, although the contradicting studies were noted to have significant limitations that question validity in the wider AKI population. For example, one study attempted to assess the diagnostic performance of FEUrea compared with FENa to distinguish between transient and persistent AKI in 99 patients hospitalized with AKI at a tertiary-care center.^12^ CKD was present in 39% of patients. In this study, a FEUrea of 35% or less and FENa of 1% or less were used to define transient AKI. The sensitivity and specificity of FEUrea were 48% and 75%, respectively, in patients not administered diuretics and 79% and 33%, respectively, in patients administered diuretics. The sensitivity and specificity of FENa were 78% and 75%, respectively, in patients not administered diuretics and 58% and 81%, respectively, in those administered diuretics. On the basis of their findings, FEUrea performed poorly in the differentiation of types of AKI, including in those on diuretics. This study had notable limitations. Most patients were nonoliguric (only 25 patients were reported as oliguric). Importantly, time between AKI diagnosis and FEUrea evaluation (*i.e.*, specimen collection) was quite prolonged (1.9±1.3 days for persistent AKI and 1.2±1.2 days for the transient AKI group). This could have significantly affected the results owing to interventions being performed, such as fluids after recognition of AKI but before FEUrea measurements. In addition, AKI in this study was defined as a 30% increase from baseline. This was likely selected for a group of patients with AKI, as for example, a rise of Cr from baseline of 2.2–2.8 would not have been considered AKI under this definition. In addition, time between the last dose of a diuretic and urine sample was only known for two-thirds of the patients.

Another relatively large observational, prospective, multicenter study in 203 intensive care unit patients showed a mean FEUrea of 41% in the transient AKI group and 32% in the persistent AKI group (*P* = 0.12).^13^ The ability of FEUrea to distinguish transient AKI from persistent AKI was poor, with the area under the receiver-operating curve being 0.59. Using a FEUrea cutoff of 35%, sensitivity was reported at 63% and specificity was 54%. The results were not significantly different in subgroup analysis in patients receiving diuretics. Most of the patients had sepsis. Notable in this study was the overall poor performance of all other urinary markers assessed. In addition, unlike previous studies, AKI type (transient versus persistent) was determined solely on the grounds of time to recovery and not through any clinical criteria or comprehensive clinical assessment, which possibly affected whether patients were accurately categorized.

In a recent addition, a comprehensive review of the literature and meta-analysis looked at the ability of FENa to differentiate prerenal from intrinsic AKI.^14^ This was the first meta-analysis to look at this issue. The analysis included 19 studies and a total of 1287 patients. Most of the included studies used a threshold of 1% to differentiate intrinsic from prerenal AKI. At this threshold for all the included patients in these studies, the ability of FENa to differentiate prerenal from intrinsic AKI had a pooled sensitivity of 90% and specificity of 82%. This corresponded to a positive predictive value of 85%. When the analysis was restricted to studies that included patients with CKD or patients on diuretics, the pooled sensitivity and specificity dropped to 83% and 66%, respectively. In eight studies with 264 patients who were oliguric, but had no CKD and did not receive any diuretic therapy, the pooled sensitivity and specificity increased to 95% and 91%, respectively. It was also noted that the finding of FENa <1% was associated with a relatively high negative predictive value of 82% in patients on diuretics and 88% in patients on diuretics and those with CKD, indicating that a low FENa despite the use of diuretics and presence of CKD is likely consistent with prerenal AKI.

The rationale of differentiation between prerenal and intrinsic AKI is to allow for identifying those who would benefit from appropriate interventions in the setting of decreased effective circulatory volume. In addition, it may lead to preventing aggressive and inappropriate IV fluids with its related complications in those with intrinsic AKI. FENa is criticized for being an imperfect marker, given multiple exceptions in causative etiologies, and that it is mostly applicable in oliguric patients. However, the only way FENa should be used is in conjunction with a thorough clinical assessment along with other available diagnostic tools. This approach applies to any test that we use in clinical practice.

When should FENa be used? On the basis of the data presented, in a hospitalized patient with AKI, FENa performs best if used in those who are oliguric, those who have not received recent diuretics, and who do not have CKD. In this setting, the ability of FENa to differentiate prerenal from intrinsic AKI is high and can guide the clinician in management. As a part of the clinical assessment, patients who are suspected to have urinary obstruction, have other manifestations suggestive of GN, or seemingly have a drug reaction should be managed accordingly, and FENa use is discouraged. In patients who have recently received diuretics, FEUrea is a more appropriate test.

In summary, FENa is a rapid, relatively inexpensive, and rather noninvasive test that helps guide clinicians in the management of AKI. Like any other diagnostic test, it should be used in conjunction with the overall assessment and other clinical tools, and the way it is interpreted and its limitations should be well-known to the evaluating clinician. Until we have a better marker to differentiate prerenal from intrinsic AKI, FENa should continue to be a part of our armamentarium.

## References

[1] Espinel CH. The FENa test. Use in the differential diagnosis of acute renal failure. JAMA. 1976;236(6):579–581. doi:10.1001/jama.236.6.579 947239

[2] Miller TR Anderson RJ Linas SL, . Urinary diagnostic indices in acute renal failure: a prospective study. Ann Intern Med. 1978;89(1):47–50. doi:10.7326/0003-4819-89-1-47 666184

[3] Tankhiwale SR Ungratwar KP. Diagnostic evaluation of urinary indices in acute renal failure. J Assoc Physicians India. 1987;35(8):557–559. 3693308

[4] Brown MA Trew PA Smart RC. A simple aid to the differential diagnosis of oliguria. Aust N Z J Med. 1983;13(6):608–612. doi:10.1111/j.1445-5994.1983.tb02614.x 6586151

[5] Espinel CH Gregory AW. Differential diagnosis of acute renal failure. Clin Nephrol. 1980;13(2):73–77. 7363517

[6] Steiner RW. Interpreting the fractional excretion of sodium. Am J Med. 1984;77(4):699–702. doi:10.1016/0002-9343(84)90368-1 6486145

[7] Zarich S Fang LS Diamond JR. Fractional excretion of sodium. Exceptions to its diagnostic value. Arch Intern Med. 1985;145(1):108–112. doi:10.1001/archinte.145.1.108 3970621

[8] Kaplan AA Kohn OF. Fractional excretion of urea as a guide to renal dysfunction. Am J Nephrol. 1992;12(1-2):49–54. doi:10.1159/000168417 1415365

[9] Carvounis CP Nisar S Guro-Razuman S. Significance of the fractional excretion of urea in the differential diagnosis of acute renal failure. Kidney Int. 2002;62(6):2223–2229. doi:10.1046/j.1523-1755.2002.00683.x 12427149

[10] Diskin CJ Stokes TJ Dansby LM Radcliff L Carter TB. The comparative benefits of the fractional excretion of urea and sodium in various azotemic oliguric states. Nephron Clin Pract. 2009;114(2):c145–c150. doi:10.1159/000254387 19887835

[11] Dewitte A Biais M Petit L, . Fractional excretion of urea as a diagnostic index in acute kidney injury in intensive care patients. J Crit Care. 2012;27(5):505–510. doi:10.1016/j.jcrc.2012.02.018 22520491

[12] Pépin MN Bouchard J Legault L Ethier J. Diagnostic performance of fractional excretion of urea and fractional excretion of sodium in the evaluations of patients with acute kidney injury with or without diuretic treatment. Am J Kidney Dis. 2007;50(4):566–573. doi:10.1053/j.ajkd.2007.07.001 17900456

[13] Darmon M Vincent F Dellamonica J, . Diagnostic performance of fractional excretion of urea in the evaluation of critically ill patients with acute kidney injury: a multicenter cohort study. Crit Care. 2011;15(4):R178. doi:10.1186/cc10327 21794161 PMC3387621

[14] Abdelhafez M Nayfeh T Atieh A, . Diagnostic performance of fractional excretion of sodium for the differential diagnosis of acute kidney injury: a systematic review and meta-analysis. Clin J Am Soc Nephrol. 2022;17(6):785–797. doi:10.2215/CJN.14561121 35545442 PMC9269645

